# Profile for Brain Disease Research Infrastructure for Data Gathering and Exploration (BRIDGE) Platform

**DOI:** 10.14336/AD.2024.1432

**Published:** 2024-12-07

**Authors:** Sujung Oh, Hee-Young Sohn, Junwoo Seo, Eunjee Kang, Jae Kyung Park, So Young Moon, Hee Jin Kim, Na-Yeon Jung, Sun Min Lee, Bo Kyoung Cheon, Hyemin Jang, Sung Hoon Kang, Sarang Kang, Kyu Yeong Choi, Sang-Won Yoo, Yun Joong Kim, Juhee Cho, Eun-Joo Kim, Sang Won Seo, Kun Ho Lee, Joong-Seok Kim, Young Ho Koh, Chi-Hun Kim, Munjin Kwon, Danbee Kang

**Affiliations:** ^1^Department of Clinical Research Design and Evaluation, SAIHST, Sungkyunkwan University, Seoul, Korea.; ^2^Center for Clinical Epidemiology, Samsung Medical Center, Seoul, Korea.; ^3^Department of Chronic Disease Convergence Research, Division of Brain Disease Research, Korea National Institute of Health, Cheongju, Korea.; ^4^Department of Neurology, Ajou University School of Medicine, Suwon, Korea.; ^5^Department of Neurology, Samsung Medical Center, Sungkyunkwan University School of Medicine, Seoul, Korea.; ^6^Department of Neurology, Pusan National University Yangsan Hospital, Pusan National University School of Medicine, Yangsan, Korea.; ^7^Alzheimer's Disease Convergence Research Center, Samsung Medical Center, Seoul, Korea.; ^8^Neuroscience Center, Samsung Medical Center, Seoul, Korea.; ^9^Department of Neurology, Seoul National University Hospital, Seoul National University College of Medicine, Seoul, Korea.; ^10^Department of Neurology, Korea University Guro Hospital, Korea University College of Medicine, Seoul, Korea.; ^11^Gwangju Alzheimer's disease and Related Dementia Cohort Research Center, Chosun University, Gwangju, Korea.; ^12^Department of Neurology, College of Medicine, The Catholic University of Korea, Seoul, Korea.; ^13^Department of Neurology, Yonsei University College of Medicine, Seoul, Korea.; ^14^Department of Neurology, Yongin Severance Hospital, Yonsei University Health System, Yongin, Korea.; ^15^Department of Neurology, Pusan National University Hospital, Pusan National University School of Medicine and Medical Research Institute, Busan, Korea.; ^16^Department of Biomedical Science, Chosun University, Gwangju, Korea.; ^17^Department of Neural Development and Disease, Korea Brain Research Institute, Daegu, Korea.; ^18^Department of Neurology, SAIHST, Sungkyunkwan University, Seoul, Korea.

**Keywords:** Cohort profile, brain disease, platform, dementia, Parkinson's disease

## Abstract

Brain diseases complexity have necessitated advanced research platforms for better understanding, treatment, and prevention strategies. However, existing brain disease registries face limitations such as incomplete variable sets, lack of standardization, insufficient linkage to external databases, absence of integrated platforms for comprehensive data collection, and lack of continuity. To address these challenges, the Korea National Institute of Health initiated the Brain disease Research Infrastructure for Data Gathering and Exploration (BRIDGE), a national prospective platform designed to overcome the shortcomings of current registries. The BRIDGE platform includes a Longitudinal Study of Early onset dementia And Family members (LEAF) cohort, a Longitudinal/cohort Study of Patients with Late Onset Dementia (LLOD) cohort, a community-based cohort study of High-risk individuals for Dementia (COHD) cohort, and a Longitudinal Study of Patients with Parkinson’s Disease (LoPD) cohort. The standardized variables included sociodemographic variables, health behaviors, medical history, activities of daily living, behavioral, and psychological problems, cognitive function, disease-related symptoms, quality of life (QoL), sleep, depression scale, caregiver burden, physical health, blood tests, olfactory function testing, orthostatic blood pressure changes, genetic testing, nerve conduction studies, and neuroimaging. In addition, the BRIDGE platform will be linked to the Korean National Health Insurance Service (K-NHIS) database. By addressing gaps in data collection, standardization, and considering a wide range of impacts, the BRIDGE database offers new pathways for understanding and combating complex brain conditions. As the project progresses, it has the potential to significantly influence scientific understanding and policymaking in the field of brain health.

## INTRODUCTION

Brain diseases such as Alzheimer's disease (AD), Parkinson's disease (PD), and stroke, have significant burdens on global health and economies [1]. Brain diseases cause progressive cognitive impairment, physical disabilities, and increased mortality, affecting millions of people worldwide. The costs of brain disease care are high, with an estimated annual cost of $32,865 per person in high-income countries. Effective treatments and preventive strategies are needed to manage brain diseases. However, the high cost and time required for conducting clinical trials is a major barrier to drug development for brain diseases.

The construction of brain disease registries can improve clinical research. Such registries have the potential to revolutionize approaches to brain disease research by simplifying patient recruitment for clinical trials, facilitating longitudinal studies to reveal disease progression, developing clues for new drugs, building evidence for public health policies, and encouraging collaborations across research institutions [2, 3]. However, current registries face significant challenges. They lack comprehensive variables, suffer from non-standardized data collection that hinders comparative analysis, fail to link with external databases due to inadequate informed consent processes, and do not provide integrated data collection, monitoring, and sharing. In particular, research databases exist in isolation with no practical avenue for sharing or pooling medical data into high dimensional datasets that can be efficiently compared across databases. There is increasing pressure on the research community to make data more findable, accessible, interoperable, and reusable [4], pushing beyond individual researchers’ desire to share their data [5].

Although the platform is well-structured, the cohorts were collected using a variety of methodologies, with data from each cohort later combined. Additionally, no external follow-up data was linked (Supplementary Table 1). Thus, the Korea National Institute of Health (KNIH) initiated the Brain disease Research Infrastructure for Data Gathering and Exploration (BRIDGE), a national prospective brain disease platform, to address these challenges. BRIDGE aims to serve as a comprehensive, standardized, and integrative resource for brain disease research, overcoming the limitations of existing registries. BRIDGE ensures extensive variable collection, standardization for comparability, linkage to external databases, and continuous government-backed support. In this paper, we describe the methods used to develop the BRIDGE platform, its capabilities, and its cohort profile, including the characteristics of enrolled participants.

## MATERIALS AND METHODS

### Data sources for the BRIDGE platform

The BRIDGE platform is a large-scale informatics platform designed to support the collection, storage, federation, sharing, and analysis of different data types across several brain disorders, to help understand common underlying causes of brain dysfunction and develop novel approaches to treatment. The strength of the BRIDGE platform is that it prospectively collects data after confirmation of variables and standardization with the entire cohort.

**Figure 1.**
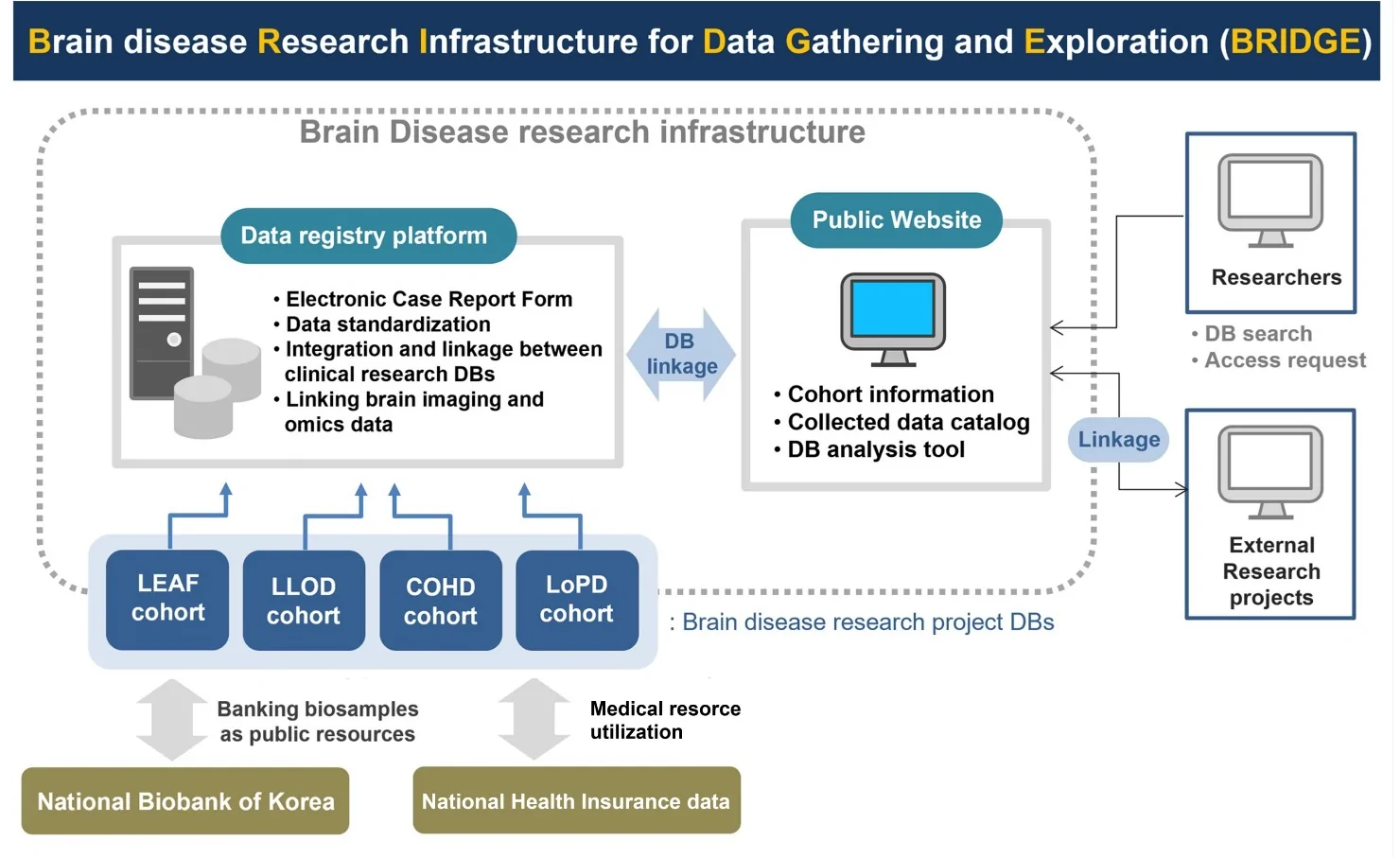
**Schematic representation of BRIDGE platform infrastructure**. BRIDGE, Brain disease Research Infrastructure for Data Gathering and Exploration; LEAF, the Longitudinal Study of Early onset dementia And Family members cohort; LLOD, the Longitudinal/cohort Study of Patients with Late Onset Dementia cohort; COHD, the Community-based cohort study of High-risk individuals for Dementia cohort; LoPD, the Longitudinal Study of Patients with Parkinson’s Disease BRIDGE platform provides a web-based electronic case report forms (eCRFs) input system for LEAF, LLOD, COHD and LoPD cohort studies, allowing participating centers in each cohort to easily access online and register the collected data. It maintains high-quality data by monitoring, making improvements of eCRF system for efficiency and accuracy, and cleansing according to prepared data management plans (DMPs). The bio-resources collected in each cohort would be shared with researchers for brain disease research by National Biobank of Korea, and data information could be found on the public website. BRIDGE platform database, built through data standardization and harmonization, could be linked not only between each cohort but also with external research projects.

The BRIDGE platform consists of four parallel and complementary prospective cohorts. Prior to the recruitment of patients, the four cohorts collaborated to plan the collection of variables and methods, with the aim of integrating them all within the BRIDGE platform (Supplementary Table 2). These cohorts include clinical data, imaging data, and biospecimens from patients who have been diagnosed with or are at high risk of developing brain disorders. The four cohorts are: the Longitudinal Study of Early onset dementia And Family members (LEAF) cohort, the Longitudinal/cohort Study of Patients with Late Onset Dementia (LLOD) cohort, the Community-based cohort study of High-risk individuals for Dementia (COHD) cohort, and the Longitudinal Study of Patients with Parkinson’s Disease (LoPD) cohort (Fig. 1). There are a total of 70 centers participating in the LEAF, LLOD, COHD, and LoPD cohorts.

The LEAF cohort consists of patients and family members with early-onset dementia, defined as patients who had symptoms before the age of 65including frontotemporal dementia (FTD), ADCI and other early onset neurodegenerative diseases. The LLOD cohort includes patients aged 65 and older with late-onset dementia, specifically Alzheimer's disease related cognitive impairment (ADCI), vascular cognitive impairment (VCI), and Lewy body disease (LBD). The COHD cohort includes community-based normal healthy participants and patients with Alzheimer's disease or mild cognitive impairment (MCI) who are over 55 years of age. The LoPD cohort includes patients with Parkinson’s disease who visited movement disorder clinics. Each cohort is followed-up every 1 or 2 years depending on the purpose of the study (Supplementary Table 1). All centers participating in each cohort project received Institutional Review Board approval and followed the principles outlined in the Declaration of Helsinki.

### Data collection and management

**Figure 2.**
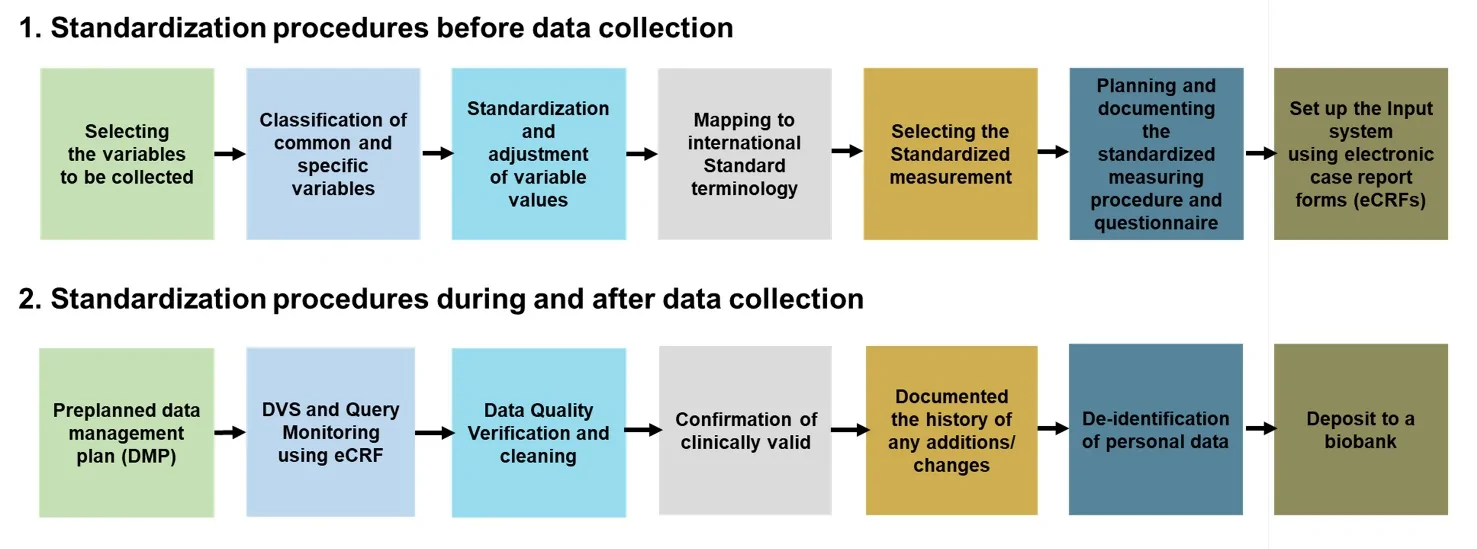
**The process to develop BRIDGE platform**. BRIDGE, Brain disease Research Infrastructure for Data Gathering and Exploration.

Figure 2 describes the processes for collecting and managing the data in the BRIDGE platform. The first step was to select variables for data. We reviewed existing cohort data on brain diseases (Supplementary Table 1). We then used the Delphi method to obtain expert opinions on the importance and feasibility of collecting clinical information from each cohort. A total of 24 experts participated in this survey. Experts were consulted on the importance and feasibility of using a 5-point Likert scale. The results were summarized, and an online expert meeting was held to review them. This process led to the identification of the final common domains and items.

Second, considering the importance and feasibility of data collection, common domains and items were identified and tailored to the specific needs of each cohort. Cohort specific items were selected when there was a desire to collect information beyond the chosen items, a need for more detailed data collection, or a preference for data collection using disease-specific tools.

Third, although items may seem to be identical across studies, their definitions may vary depending on the perspective, which can lead to inconsistent data collection. Therefore, clear definitions for the common items were established to ensure precision.

Fourth, mapping clinical items to international standard terminologies enables computers to process them using unique identifiers (IDs) and ensures a clear understanding of what the items represent. Therefore, we mapped the selected common items to international standard terminologies.

Fifth, experts in neurology, epidemiology, biostatistics, and other relevant fields conducted comprehensive reviews of existing instruments and selected internationally accepted measures (Table 1) that are validated and reliable for assessing various aspects of brain diseases. Adapting and validating instruments in the Asian population is crucial for improving linguistic accuracy, cultural sensitivity, and relevance, especially when instruments were originally developed in different cultural and linguistic contexts.

**Table 1:** Data items collected for the BRIDGE platform.

Items	Common items	By cohort				Relation to International Standards
		LEAF	LLOD	COHD	LoPD	
**Sociodemographic variables**						
Gender	O	O	O	O	O	184100006 |Patient sex (observable entity)|
Date of birth	O	O	O	O	O	184099003 |Date of birth (observable entity)|
Age	O	O	O	O	O	424144002 |Current chronological age (observable entity)|
Address	O	O	O	O	O	184097001 |Patient address (observable entity)|
Living arrangement	O	O	O	O	O	224209007 |Residence and accommodation circumstances (observable entity)|
Cohabitant	O	O	O	O	O	224130005 |Household composition (observable entity)|
Caregiver	O	O	O	O	O	
Type, age, frequency of meetings						46527-8 |Primary caregiver [OASIS]|, 443443002 |Age of caregiver (observable entity)|
Highest level of education	O	O	O	O	O	224293004 |Education received in the past (observable entity)|
Literacy	O	O	O	O	O	
Job	O	O	O	O	O	
Current employment status, current occupation, longest occupation						88381-9 |Do you currently have a job or do any unpaid work outside your home [IPAQ]|, 85658-3 |Occupation [Type]|, 21843-8 |History of Usual occupation|
Hand laterality	O	O	O	O	O	57427004 |Handedness (observable entity)|
Marital status	O	O	O	O	O	125680007 |Marital status (observable entity)|
Average monthly income	O	O	O	O	O	77244-2 |Total combined household income range in last year|
**Health behaviors**						
Smoking	O	O	O	O	O	63581-3 |Smoked at least 100 cigarettes in entire life|, 63582-1 |Do you now smoke cigarettes every day, some days, or not at all?|, 64218-1 |How many cigarettes do you smoke per day now [PhenX]|, 63640-7 |How many cigarettes per day do/did you smoke?|, 63632-4 |About how long has it been since you COMPLETELY quit smoking cigarettes?|
Drinking	O	O	O	O	O	63633-2 |In your entire life, have you had at least 1 drink of any kind of alcohol, not counting small tastes or sips [AUDADIS-IV]|, 63634-0 |About how old were you when you first started drinking, not counting small tastes or sips of alcohol [PhenX]|, 68518-0 |How often do you have a drink containing alcohol|, 68519-8 |How many standard drinks containing alcohol do you have on a typical day|
Physical activity	O	O	O	O	O	88382-7 |During the last 7 days, on how many days did you do vigorous physical activities like heavy lifting, digging, heavy construction, or climbing up stairs as part of your work for at least 10M at a time [IPAQ]|, 88383-5 |How much time did you usually spend on one of those days doing vigorous physical activities as part of your work during the last 7 days [IPAQ]|, 88384-3 |During the last 7 days, on how many days did you do moderate physical activities like carrying light loads as part of your work, not including walking, greater than 10 minutes at a time [IPAQ]|, 88385-0 |How much time did you usually spend on one of those days doing moderate physical activities as part of your work during the last 7 days [IPAQ]|, 88391-8 |During the last 7 days, on how many days did you bicycle for at least 10 minutes at a time to go from place to place [IPAQ]|, 88393-4 |During the last 7 days, on how many days did you walk for at least 10 minutes at a time to go from place to place [IPAQ]|, 88392-6 |How much time did you usually spend on one of those days to bicycle from place to place during the last 7 days [IPAQ]|, 88394-2 |How much time did you usually spend on one of those days walking from place to place during the last 7 days [IPAQ]|, 88405-6 |During the last 7 days, on how many days did you do vigorous physical activities like aerobics, running, fast bicycling, or fast swimming in your leisure time for at least 10 minutes at a time [IPAQ]|, 88406-4 |How much time did you usually spend on one of those days doing vigorous physical activities in your leisure time during the last 7 days [IPAQ]|, 88407-2 |During the last 7 days, on how many days did you do moderate physical activities like bicycling at a regular pace, swimming at a regular pace, and doubles tennis in your leisure time for at least 10 minutes at a time [IPAQ]|, 88408-0 |How much time did you usually spend on one of those days doing moderate physical activities in your leisure time during the last 7 days [IPAQ]|
Digital device / smartphone use ability		O	O			
Oral health			O			
Sleep disorders						
K-PSQI [17, 18]		O	O	O		Korean validation version for PSQI
RBDQ-KR [19]		O				Korean validation version for RBD
RBDSQ-K [20]					O	Korean validation version for RBDSQ
KESS [21]					O	Korean validation version for ESS
K-PDSS-2 [22]					O	Korean validation version for PDSS
Nutrition and diet						
MNA [23]		O	O			Korean validation version for MNA
MDA		O				Korean validation version for MDA
NQ-E [24]		O				Korean validation version for NQ-E
Coffee/tea			O			
COVID-19 questionnaire				O		
**Medical history**						
Disease history	O	O	O	O	O	
Hypertension, stoke, heart, diabetes, hyperlipidemia, neuropsychiatric						11349-8 |History of Past illness|
Family history	O	O	O	O	O	
Dementia, Parkinson’s disease						8670-2 |History of family member diseases|
Family tree (doctor)		O				
Diagnosis	O	O	O	O	O	439401001 |Diagnosis (observable entity)|, 63931-0 |Date of diagnosis|
Neurological test (consultation)		O				
Evaluation details (doctor)		O				
Symptom Related Information (doctor)		O				
Medicine usage list		O				
**Activities of daily living**						
K-IADL[25, 26]		O	O	O		Korean validation version for IADL
Bathel ADL Index [27]		O	O			Korean validation version for Bathel ADL index
**Behavior and psychological problems**						
Behavior problems						
K-NMSS [28]			O		O	Korean validation version for NMSS
FBI		O				Korean validation version for FBI
K-IRI		O				Korean validation version for IRI
Revised Self-Monitoring Scale [29]		O				
Psychological problems						
STR short ver.[30]			O			
BAI [31]			O			Korean validation version for BAI
K-AD8 [32, 33]			O			Korean validation version for AD8
K-GAI [34]		O				Korean validation version for GAI
BEPSI		O				Korean validation version for BEPSI
SGDS-K [35]		O				Korean validation version for SGDS
GDS-K [35-37]				O		Korean validation version for GDS
**Cognitive function**						
K-MMSE [38]	O	O	O	O	O	Korean validation version for MMSE
CDR5 [39]	O	O	O	O	O	Korean validation version for CDR
SNSB [40]	O	O	O	O	O	Korean Adaptation of the Revised CERAD Questionnaire
K-ECog [41, 42]		O	O			Korean validation version for ECog
Cognitive/social activity		O	O			
KDSQ		O		O		MMSE Korea-specific supplemental questionnaire
K-CRIq		O				Korean validation version for CRIq
FTD-CDR [43]		O				
FTD-cog score [44]		O				
K-WAB [45]		O				Korean validation version for WAB
ADAS-Cog [46]		O				
FEDAS		O				
CCI		O				
MoCA-K [47]					O	Korean validation version for MoCA
SMCQ [48]				O		
**Disease related symptoms**						
K-PDQ-39 [49]					O	Korean validation version for PDQ-39
K-MADRS					O	Korean validation version for MADRS
Parkinson’s disease information about patient					O	
MDS-UPDRS [50]					O	
**Quality of life**						
EQ-5D	O	O	O	O	O	
**Examination**						
Neuroimaging						
MRI	O	O	O	O	O	439272007 |Date of procedure (observable entity)|
PET	O	O	O	O	O	439272007 |Date of procedure (observable entity)|
FP-CIT analysis data					O	
Blood test	O	O	O	O	O	
WBC, RBC, Hb, Hct, PLT, ALT, AST, BUN, Cr, Glucose, HbA1C, HDL cholesterol, total cholesterol, LDL cholesterol, TG, TSH, FT4, Folate, Vitamin B12					
Genetic type	O	O	O	O	O	
APOE						34438-2 |Apolipoprotein E phenotype [Identifier] in Blood|
Anthropometric	O	O	O	O	O	
Weight, height, body mass index						8308-9 |Body height --standing|, 8306-3 |Body height --lying|, 3141-9| Body weight Measured|, 39156-5 |Body mass index (BMI) [Ratio]|
Vital sign	O	O	O	O	O	
Measurement equipment, measurement arm, measurement conditions, blood pressure, purse						41901-0 |Type of Blood pressure device|, 41904-4 |Blood pressure measurement site|, 8480-6 |Systolic blood pressure|, 96608-5 |Systolic blood pressure unspecified time mean|, 8462-4 |Diastolic blood pressure|, 96609-3 |Diastolic blood pressure unspecified time mean|, 8867-4 |Heart rate|
**Caregivers**						
Behavior						
Caregiver medical use		O				
Psychological problems						
BFI-K-10		O				Korean validation version for BFI
K-DUREL and K-DSES		O				Korean validation version for DUREL and DSES
ZBI [51]		O				Korean validation version for ZBI
Social support / systems related to caregiving		O				
Korean SF-36 Health survey [52]		O				Korean validation version for SF-36
KBDI-II		O				Korean validation version for BDI-II
CBI [53]					O	Korean validation version for CBI
Cognitive assessment						
CGA-NPI [54]		O	O			
CAS-K		O				Korean validation version for CAS

ADAS-Cog, Alzheimer Disease Assessment Scale-cognitive subscale; ALT, Alanine Aminotransferase; APOE, Apolipoprotein-E; AST, Aspartate Aminotransferase; BAI, Beck Anxiety Inventory; BEPSI, Brief Encounter Psychosocial Instrument; BFI-K-10, Big Five Inventory-Korean ver; BUN, Blood Urea Nitrogen; CAS-K, Korean Version of Caregiver Activity Survey; CBI, Korean version of the Caregiver Burden Inventory; CCI, Cognitive complaint interview; CDR, Clinical Dementia Rating; CGA-NPI, Caregiver-Administered Neuropsychiatric Inventory; Cr, Creatinine; FBI, Frontal Behavioral Inventory; FEDAS, Frontal Executive dysfunction/Disinhibition/Apathy Scale; FP-CIT analysis data, Fluoropropyl-CIT; FTD, Frontotemporal Dementia; FTD-CDR, FTD Clinical Dementia Rating; FTD-cog, cognitive test battery for FTD; FT4, Free Thyroxine 4; GDS-K, Korean version of the Geriatric Depression Scale; Hb, Hemoglobin; HDL cholesterol, High-Density Lipoprotein cholesterol; Hct, Hematocrit; HbA1c, Hemoglobin A1c; K-AD8, Korean Version of the Alzheimer disease 8 Informant Interview; KBDI-II, Korean Version of Beck-II Depression Inventory; K-CRIq, Korean version of Cognitive Reserve Index questionnaire; K-DSES, Korean Versions of the Daily Spiritual Experience Scale; KDSQ, Korean Dementia Screening Questionnaire; K-DUREL, Korean Versions of the Duke University Religion Index; K-ECog, Korean-Everyday Cognition; KESS, Korean version of the Epworth sleepiness scale; K-GAI, Korean Geriatric Anxiety Inventory; K-IADL, Korean Instrumental Activities of Daily Living; K-IRI, Korean version of Interpersonal Reactivity Index; K-MADRS, Korean Version of the Montgomery-Asberg Depression Rating Scale; K-MMSE, Korean Mini-mental State Examination; K-NMSS, Korean-Version of the Nonmotor Symptoms Scale; K-PDQ-39, Korean Version of the 39-Item Parkinson’s Disease Questionnaire; K-PDSS-2, Korean Version of Parkinson’s Disease Sleep Scale-2; K-PSQI, Korean version of the Pittsburgh Sleep Quality Index; K-WAB, Korean Version of the Western Aphasia Battery; LDL cholesterol, Low-Density Lipoprotein cholesterol; MDA, Mini Dietary Assessment; MDS-UPDRS, Korean Version of the Movement Disorder Society-Unified Parkinson’s Disease Rating Scale; MNA, Mini Nutritional Assessment; MoCA-K, Korean version of Montreal Cognitive Assessment; MRI, Magnetic Resonance Imaging; NQ-E, Nutrition Quotient for Korean elderly; PET, Positron Emission Tomography; PLT, Platelet; RBC, Red Blood Cell; RBDQ-KR, REM Sleep Behavior Disorder Questionnaire-Korean; RBDSQ-K, Korean version of the REM sleep behavior disorder screening questionnaire; SGDS-K, Korean version of the short form of Geriatric Depression Scale; SMCQ, Subjective Memory Complaints Questionnaire; SNSB, Seoul Neuropsychological Screening Battery; STR, Stress Questionnaire for KNHANES short ver; TG, Triglyceride; TSH, Thyroid Stimulating Hormone; WBC, White Blood Cell; ZBI, Korean Versions of the Zarit Burden Interview

Sixth, we planned and documented how to perform measurements according to standardized procedures. We produced a manual to standardize equipment and supplies used for physical measurements, vital signs, blood tests, and other methods in the Korea National Health and Nutrition Examination Survey (KNHANES). Standardizing equipment across participating institutions in the BRIDGE platform is challenging. Therefore, we adopted methods used by the participating institutions and collected information on sample collection, analytical methods, equipment, reagents, reference ranges, units, and quality control methods for each test item based on the data from each institution.

Seventh, we developed a web-based input system using electronic case report forms (eCRFs) for the platform. We integrated a data verification system (DVS) into the eCRF to improve the data quality and integrity. The DVS conducted based on predefined rules and logic checks. Alerts and queries are automatically generated when inconsistent or implausible values are entered, allowing for prompt review and correction when necessary immediate corrections. DVS helps to enhance data quality and minimize delays in the research workflow. We carefully reviewed and organized this system to ensure a high standard of data accuracy and reliability, thereby strengthening the overall robustness of our eCRF infrastructure. The BRIDGE registry platform was routinely visualized using a series of dashboards. Dashboards help track progress and examine the results of various recruitment efforts, and customized dashboards are available for various sub-studies.

### Measurements

Finally, 164 items in 10 domains were confirmed as common data set (CDS) variables for all cohorts. In addition, cohort-specific variables for the LEAF, LLOD, COHD, and LoPD cohorts included 2,380, 1,460, 1,300, and 1,270 items, respectively (Table 1).

#### Clinical data and cognitive function test

Clinical data including type of disease, age of symptom onset, year at diagnosis, duration of illness, and history of medication usage were collected by trained researchers from electronic health records (EMRs). All participating centers were required to hire at least one clinical research coordinator (CRC), whose responsibility was the collection and input of data into the eCRF for the BRIDGE platform. The trained researchers include a multidisciplinary team comprising healthcare professionals, such as physicians and CRC, as well as data managers with expertise in handling electronic health records (EHRs). These individuals receive additional training specific to the BRIDGE platform, including the standardized collection and management of clinical, cognitive, and other cohort-specific data using the eCRF system. Common medical history items included hypertension, stroke, diabetes, dyslipidemia, other neurological disorders, vision, hearing status, and a family history of dementia and Parkinson's disease. Pedigree charts of subjects with genetic histories were created in the LEAF. For the LEAF cohort, assessment and medical details included neurological tests, assessment details, symptom-related information, and lists of medications used.

Cognitive function was assessed using screening tools and a detailed disease assessment tool for all cohorts. Common cognitive assessment items included the Korean Mini-Mental State Examination (K-MMSE), Clinical Dementia Rating (CDR5), and the second edition of the Seoul Neuropsychological Screening Battery (SNSB-II). The Korean Everyday Cognition (K-ECOg) and cognitive/social activity questionnaires were included in the dementia cohort, including LEAF and LLOD. In contrast, the COHD and LoPD cohort included the Korean version of the Montreal Cognitive Assessment (MoCA-K) and Subjective Memory Complaints Questionnaire (SMCQ), respectively. LEAF included various types of cognitive assessment tools such as the Cognitive Complaint Interview (CCI) and the Korean version of the Cognitive Reserve Index questionnaire (CRI).

#### Questionnaires

Data on sex, date of birth, age, address, living arrangements, cohabitation, and primary caregivers (type, age, frequency of meetings, time spent, frequency of phone calls, highest level of education, years of education, literacy, current employment status, current occupation, longest occupation, marital status, hand laterality, and average monthly income) were collected. In addition, civil registration numbers were collected for links to other databases, such as public data repositories, health administration data, electronic medical records, and legacy databases.

Smoking status, alcohol consumption, physical activity, and quality of life (QoL) were assessed. Sleep quality was measured in the LEAF, LLOD, and COHD cohorts. Because digital therapeutics for dementia are expected to gain popularity, smartphone use, and nutritional and dietary data were collected as variables in the LLOD and LEAF cohorts.

Data for the LEAF, LLOD, and COHD cohorts included activities of daily living. The LEAF cohort underwent cognitive and mental health assessments and experienced daily religious activities such as praying, meditating, worshiping, attending court, or attending religious meetings. Data for the LoPD cohort included disease specific symptoms using the Korean version of the 39-item Parkinson’s Disease Questionnaire (K-PDQ-39), Korean version of the Parkinson’s Disease Sleep Scale-2 (K-PDSS-2), Korean version of the Pittsburgh Sleep Quality Index (K-PSQI), Korean version of the Montgomery-Asberg Depression Rating Scale (K-MADRS), Parkinson's disease information about each patient, the non-motor symptoms severity scale (NMSS), and Korean version of the Movement Disorder Society-Unified Parkinson’s Disease Rating Scale (MDS-UPDRS).

In the LEAF cohort, surveys of caregivers included the Caregiver Activity Survey (CAS-K), Zarit Burden Interview (ZBI), and others related to social support/institutional support for caregiving. CAS measures the time and distress experienced by caregivers of dementia patients. The ZBI evaluates the extent of burden experienced by caregivers. Surveys related to social support and systems related to caregiving were conducted as factual investigations.

#### Physical and laboratory examinations

Common physical health assessment items included body measurements, height, weight, body mass index (BMI), vital signs, arm measurements, systolic and diastolic blood pressure, and pulse rate. Smell identification tests were performed in LoPD.

Specific items, including olfactory function testing, orthostatic blood pressure changes (supine and tilt orthostatic blood pressure results and heart rate at different times of measurement), cerebrospinal fluid, autoimmune antibodies, edematous antibodies, nerve conduction studies, and electromyography, were collected from each cohort.

Twenty-one blood tests including complete blood count (CBC), liver function tests, renal function tests, APOE genotype, and endocrine metabolism were performed in all cohorts. For LEAF there were also white blood cell tests. Whole blood samples collected from subjects were separated into plasma and serum and DNA extracted according to the standard operating procedure (SOP) of the National Biobank in Korea under the KNIH. These resources were stored frozen and transferred to the National Biobank in Korea after the project was completed. They could be provided through a review process to researchers who want to use them in brain disease research in the future.

#### Neuroimaging

All cohorts included molecular neuroimaging (MRI and PET). T1- and T2-weighted MRI and amyloid PET data were collected in LEAF, LLOD, and COHD cohorts. Each cohort also included data from ultra-high field 7-T MRI, tau PET, and FP-CIT PET for at least some patients. A separate storage space was created for brain MRI and PET scans to allow the registration of large files integrated with a separate menu in the eCRFs though the BRIDGE platform.

The BRIDGE platform employs a number of security pipelines for imaging data. The de-identification pipeline is configured to remove or replace a set of fields within the header of MRI Digital Imaging and Communication in Medicine (DICOM) files and employs a fixed set of fields to be cleared or modified. Additionally, it was designed to integrate with cohort study data. On the brain image upload screen, the data can be easily reviewed through links to clinical information.

#### Genetic data

LEAF included whole exome sequencing (WES) to gather omics data with file extensions. For LLOD, researchers collected omics data through whole-genome sequencing (WGS). COHD acquired whole-genome SNP information based on KNIH's Korean chip. LoPD-collected omics include single nucleotide variant (SNV), WES, and global diversity array (GDA) chip data. The file extensions included vcf, bam, and fastq. We are improving the BRIDGE platform so that genetic data will be linked to clinical epidemiological data of subjects.

#### Linkage to other databases

All participants in the BRIDGE platform agreed to share their data and link it to other resources via their personal identification number. Although the Korean government only allows data to be linked to public institutions with informed consent, since the BRIDGE platform is initiated and managed by KNIH, which is a public institution, it can now allow for linking with other databases, such as the Korean National Health Insurance Service (K-NHIS) database. Korea has a mandatory social insurance system with premiums based on income level rather than health status. The K-NHIS is a single insurer in Korea that covers almost the entire population and collects data on the use of medical facilities and records of prescriptions using the International Statistical Classification of Diseases and Related Health Problems, Tenth Revision (ICD-10) diagnosis codes. The KNHIS claims database contains information on demographics, medical treatments, procedures, prescription drugs, diagnosis codes, and hospital use. Vital status and cause of death were obtained from death certificates collected by Statistics Korea at the Ministry of Strategy and Finance of South Korea.[6]

### Standardization policy and data access procedures

The BRIDGE platform is being developed to support the Findable, Accessible, Interoperable, and Reusable (FAIR) Data Principles. The BRIDGE platform system architecture provides technical capabilities to support the following functions: monitor and curate data, privacy and security, and interoperable and extensible federation systems that support harmonization, integration and query across diverse data modalities and linkages to external data sources. The platform was organized and managed according to a preplanned data management plan (DMP) to monitor and curate data.

Once researchers input data, the BRIDGE platform immediately checks the data via DVS. Statisticians download all variables annually to validate the accuracy of the data. The statistician checks the quality of data based on the Data Quality Index (DQI). The DQI is a standard rule for measuring and evaluating data quality and refers to a measurement item/baseline indicator that should be managed through continuous quality checks to minimize data defects. The statistician then creates a table of characteristics, including clinically important variables, to confirm clinical validity. If logical errors are found, the statistician and researcher review the eCRF and correct them directly. They then discussed and the rule was updated in the DVS. The history of any additions/changes to DQIs or items were documented. Once all the data have been completed and checked for validity, the personal data will be de-identified for public use and subsequently deposited in a National Biobank of Korea.

In terms of privacy and security and interoperable and extensible federation system, the BRIDGE platform allows and encourages sharing the entire de-identified BRIDGE data with qualified investigators outside the BRIDGE research group, governed by a data use agreement (DUA), as well as all collaborators using BRIDGE services. The personal identification numbers (PINs) are pseudonymised during data processing to prevent direct identification of individuals. A unique study-specific identifier is generated to replace the PIN, which is stored separately in an encrypted format on a secure server. Access to the mapping key between the PIN and the study identifier is restricted to authorized personnel under strict data governance protocols. In the case of a necessity for revision of the data, the researcher is able to use the mapping key to identify the patient. After approval by the IRB to request these data, investigators can apply for a DUA using a research proposal. Researchers interested in potential collaborations can apply for the data by submitting a form that is available on the BRIDGE website (http://dementiasplatform.kr/site). A formal application must be submitted to access the data with a detailed research proposal consisting of a title, authors, research questions, a brief scientific background, a list of required variables, and proposed statistical analyses. The KNIH Study Steering Committee reviews all proposals, and a final decision on the use of the data is provided.

### Statistical analyses

Characteristics of study participants in four cohorts were compared using ANOVA for continuous variables and χ2 tests for categorical variables. If the continuous variable was not normally distributed, the Kruskal-Wallis test was used.

To assess differences between the BRIDGE cohorts and the general population, we used data from the Korea National Health and Nutrition Examination Survey (KNHANES) from 2019-2021. The KNHANES is a nationally representative cross-sectional study of the non-institutionalized population using a multistage cluster sampling design. We did not perform a weighted analysis because the general population representatives were selected using a matching process.

All analyses were conducted using the SAS Enterprise Guide (version 7.1; SAS Institute Inc., Cary, NC, USA) and R 4.1.2 (R Foundation for Statistical Computing, Vienna, Austria). A two-tailed p-value<0.05 was considered statistically significant.

## RESULTS

The BRIDGE platform registered 3,656 participants between May 2021 and December 2023. The average age of the participants was 69 years old, and 58.8% were female (Table 2). The LLOD cohort had the highest average age (75 years), whereas the LEAF cohort had the lowest average age (61 years) (p <0.001). The LLOD cohort had the highest proportion of females (65.3%), whereas the LoPD cohort had the lowest (47.9%) (p <0.001). The LEAF cohort had the lowest K-MMSE score and the highest Clinical Dementia Rating (CDR) score. The LOPD cohort had the lowest QoL scores.

**Table 2:** Characteristics of BRIDGE cohorts (N = 3,656).

	Longitudinal Study of Early onset dementia And Family members (LEAF) cohort	Longitudinal/cohort Study of Patients with Late Onset Dementia (LLOD) cohort	Community-based cohort study of High-risk individuals for Dementia (COHD) cohort	Longitudinal Study of Patients with Parkinson’s Disease (LoPD) cohort	P-value
**N**	(N = 418)	(N = 752)	(N = 1,702)	(N = 784)	
**Sex, female (%)**	255 (61.7)	430 (65.3)	1,105 (64.9)	360 (47.9)	<0.001
**Age, years**	61.00 (7.04)	75.01 (6.15)	73.11 (5.94)	67.74 (9.35)	<0.001
**Married (%)**	374 (91.7)	459 (69.8)	1,339 (78.7)	666 (89.3)	<0.001
**Residence, house (%)**	404 (98.5)	658 (100.0)	1,702 (100.0)	743 (99.6)	<0.001
**Education (%)**					
Not educated	13 (3.2)	61 (9.3)	146 (8.6)	34 (4.5)	<0.001
≤High school graduate	271 (65.9)	465 (70.7)	1,115 (65.5)	483 (64.3)	
≥University	127 (30.9)	130 (19.8)	441 (25.9)	234 (31.2)	
**Employee/self-business (%)**	80 (19.5)	105 (16.0)	496 (29.1)	267 (35.7)	<0.001
**Hand laterality (%)**					
Right-handed	382 (92.9)	592 (90.0)	1,610 (94.6)	704 (94.4)	<0.001
Left-handed	8 (1.9)	14 (2.1)	18 (1.1)	16 (2.1)	
Both handed	21 (5.1)	31 (4.7)	74 (4.3)	26 (3.5)	
**Income (USD) (%)**					
<3K	124 (30.6)	261 (39.7)	1,110 (65.2)	247 (33.2)	<0.001
>3K	97 (24.0)	104 (15.8)	472 (27.7)	147 (19.7)	
Unknown	184 (45.4)	293 (44.5)	120 (7.1)	351 (47.1)	
**BMI, kg/m^2^ (SD)**	23.41 (3.52)	24.22 (4.05)	24.70 (3.14)	24.17 (3.16)	<0.001
**Smoke Current State (%)**					
Never	274 (68.8)	490 (76.1)	1,284 (75.4)	482 (64.6)	<0.001
Past	94 (23.6)	129 (20.0)	386 (22.7)	239 (32.0)	
Current	30 (7.5)	25 (3.9)	32 (1.9)	25 (3.4)	
**Drinking, yes (%)**	256 (64.5)	339 (52.7)	1,019 (59.9)	513 (68.8)	<0.001
**Physical activity, METs min/week**	2,520 (1,440 - 4,680)	2,520 (1,280 - 5,100)	2,880 (1,680 - 4,920)	2,640 (1,680 - 5,180)	0.075
**Comorbidity (%)**					
Hypertension	139 (34.9)	357 (55.0)	790 (46.4)	323 (43.4)	<0.001
Stroke	24 (6.0)	32 (4.9)	9 (0.5)	23 (3.1)	<0.001
Cardiovascular disease	38 (9.6)	117 (18.1)	260 (15.3)	94 (12.6)	0.001
Diabetes	62 (15.6)	178 (27.5)	354 (20.8)	147 (19.8)	<0.001
Dyslipidemia	116 (29.3)	303 (46.8)	751 (44.1)	255 (34.3)	<0.001
Other neuropsychiatric	79 (19.8)	89 (13.7)	80 (4.7)	90 (12.1)	<0.001
**Eyesight, normal (%)**	288 (72.7)	385 (59.4)	1,332 (78.3)	460 (61.9)	<0.001
**Hearing, normal (%)**	385 (97.2)	569 (87.9)	1,648 (96.8)	711 (95.7)	<0.001
**Family history (%)**					
Dementia	143 (35.8)	212 (33.1)	439 (25.8)	81 (10.9)	<0.001
Parkinson’s disease	17 (4.2)	30 (4.7)	57 (3.3)	53 (7.1)	0.001
**Major diagnosis (%)**					
Normal	8 (1.9)	104 (15.5)	1,128 (66.3)	1 (0.1)	<0.001
Mild cognitive impairment	63 (15.3)	341 (50.7)	503 (29.6)	2 (0.3)	
Dementia	342 (82.8)	227 (33.8)	70 (4.1)	1 (0.1)	
Parkinson’s disease	0 (0.0)	0 (0.0)	1 (0.1)	709 (99.4)	
**APOE (%)**					
e2/e2	1 (0.3)	3 (0.5)	2 (0.2)	4 (0.6)	<0.001
e2/e3	23 (6.4)	44 (6.7)	83 (8.3)	75 (10.3)	
e2/e4	6 (1.7)	12 (1.8)	16 (1.6)	12 (1.7)	
e3/e3	189 (52.6)	340 (51.4)	649 (64.9)	512 (70.5)	
e3/e4	104 (29.0)	217 (32.8)	235 (23.5)	112 (15.4)	
e4/e4	36 (10.0)	45 (6.8)	15 (1.5)	11 (1.5)	
**K-MMSE score**	18.98 (7.37)	23.70 (4.57)	26.95 (3.27)	26.82 (2.97)	<0.001
**CDR score**	1.09 (0.81)	0.66 (0.37)	0.18 (0.29)	0.40 (0.29)	<0.001
**EQ5D**	0.54 (0.44)	0.59 (0.44)	0.74 (0.29)	0.35 (0.50)	<0.001

Abbreviation BMI, Body Mass Index; MET, Metabolic Equivalent; APOE, Apolipoprotein-E; K-MMSE, Korean-Mini Mental State Examination; CDR, Clinical Dementia Rating; Values were presented n (%), mean (SD) or median (interquartile range)

When compared to the age-sex-matched general population from KNHANES, patients with brain disease were less likely to be employed and had lower income than those in the general population. Additionally, patients with brain disease had significantly lower QoL than the general population (0.62 vs. 0.91, p <0.001). (Table 3).

**Table 3:** Characteristics of BRIDGE cohorts compared to the general population.

	BRIDGE	KNHNES	
**N**	(N = 3,143)	(N = 3,143)	P-value
**Sex, female (%)**	1,916 (61.0)	1,972 (62.7)	0.153
**Age, years**	69.41 (7.39)	69.37 (7.50)	0.85
**Marital status, married (%)**	2,621 (83.7)	2,244 (71.4)	<0.001
Education (%)			
Not educated	184 (5.9)	446 (14.6)	<0.001
≤High school graduate	2,105 (67.0)	1,865 (61.0)	
≥University	852 (27.1)	459 (15.0)	
Unknown	0 (0.0)	289 (9.4)	
**Occupation, employee/self-business (%)**	910 (29.0)	1,126 (36.8)	<0.001
**Income (USD) (%)**			
<3K	1,524 (48.7)	2,039 (65.6)	<0.001
>3K	774 (24.7)	1,067 (34.4)	
Unknown	831 (26.6)	0 (0.0)	
**BMI (SD)**	24.35 (3.39)	24.14 (3.15)	0.011
**Smoke Current State (%)**			
Never	2,238 (72.0)	1,966 (64.3)	<0.001
Past	765 (24.6)	731 (23.9)	
Current	107 (3.4)	362 (11.8)	
**Drinking, yes (%)**	1,929 (62.1)	2,347 (78.0)	<0.001
**Comorbidity (%)**			
Hypertension	1,354 (43.5)	1,544 (50.5)	
Stroke	79 (2.5)	141 (5.1)	
Cardiovascular disease	437 (14.1)	217 (7.8)	
Diabetes	636 (20.5)	644 (21.1)	
Dyslipidemia	1,284 (41.3)	1,197 (39.1)	
EQ5D	0.62 (0.41)	0.91 (0.13)	<0.001
Physical activity, MET-minutes/week	2,800 (1,680 - 5,040)	4,620 (3,720 - 6,500)	<0.001

Abbreviation BRIDGE, Brain disease Research Infrastructure for Data Gathering and Exploration; KNHNES, Korea National Health & Nutrition Examination Survey; BMI. Body Mass Index; MET, Metabolic Equivalent Values were presented n (%), mean (SD) or median (interquartile range)

## DISCUSSION

The BRIDGE platform was designed to provide a comprehensive, standardized, and culturally relevant dataset. All cohorts of various types of brain diseases in the platform adhered to standardized protocols. BRIDGE can merge national insurance claims data to obtain long-term health outcomes without any loss to follow-up.

Although several data repositories and platforms are available to identify and access various brain cohorts, such as the Dementias Platform UK (DPUK)[7], Global Alzheimer's Association Interactive Network (GAAIN) [8], Alzheimer's Disease Data Initiative (ADDI) [9] and the National Alzheimer’s Coordinating Center (NACC) [10], each registry or cohort in each platform reports different variables. In contrast, as the BRIDGE platform has the consensus to decide on collecting variables, all sub-cohorts on the platform collected core variables using standardized methods. The BRIDGE cohorts have also established standardized methods and processes for data standardization, harmonization, and ongoing regular quality control. This ensures the reliability and comparability of the results despite variations in data collection methods.

The BRIDGE platform comprises diverse cohorts, including early-onset dementia, late-onset dementia, Parkinson's disease, and aging. The purpose of BRIDGE is to collect data and identify unique and specific factors associated with each condition. Several cohorts of patients with brain diseases, including the Alzheimer's Disease Neuroimaging Initiative (ADNI)[11], Australian Dementia Network Registry (ADNeT Registry or Registry) [12, 13], and Alzheimer’s Network (ALZ-NET) [14], have enrolled patients with various types of dementia. However, these cohorts often focus primarily on a single disease spectrum, which may inadvertently combine factors common to other neurodegenerative diseases with those unique to dementia. Identifying specific markers of dementia is a complex task due to the overlap of symptoms and factors among various brain diseases. The BRIDGE platform aims to mitigate these issues by gathering data from a wide range of patients with different but related neurological conditions. Researchers can compare and contrast data across different groups by identifying overlapping factors and focusing on specific characteristics of dementia. This will help identify specific markers of dementia, which is crucial for developing accurate diagnostic tools, understanding disease progression, and creating targeted treatments.

Furthermore, the variables were chosen based on literature review and expert opinion to predict future trends from diagnosis to survivors. Collecting a wide range of data can help to understand the broader impact of brain diseases on an individual's life. This aspect may not have been extensively covered in previous cohorts. The BRIDGE data also include the QoL of caregivers and patients and provide a holistic view of the impact of brain diseases, a critical aspect that may have received less attention in other cohorts. In our cohort profiles, patients with brain disease have significantly lower QoL compared to the age-sex matched general population, consistent with previous studies [15, 16]. However, according to a systematic review [15, 16], limitations were identified in previous studies, including the use of different QoL measures [15, 16]. In addition, previous studies did not analyze certain factors related to QoL, such as gender, disease duration, disease severity, health care system, and medication treatment, due to insufficient data [15, 16]. Therefore, BRIDGE data can be used to evaluate the QoL of patients with different brain diseases as well as the general population using the same instrument and to identify different factors associated with QoL. Clinicians can use BRIDGE platform data to develop targeted interventions to improve QoL in patients with dementia and Parkinson's disease, who appear to be particularly affected.

BRIDGE data can be merged with existing national databases, such as the KNHANES database. Using these data, we can evaluate the impacts of brain disease on QoL and conduct cost-effectiveness analyses or other research using the general population for comparison. BRIDGE data can also be merged with the K-NHIS database. This integration is expected to result in more efficient data management compared to previous cohorts. Linked claims data can effectively capture long-term health outcomes without loss to follow-up.

In conclusion, the BRIDGE platform is a major step towards combating brain diseases. By addressing gaps in data collection and standardization and considering a wide range of brain disease impacts, BRIDGE data offers new pathways for understanding and combating these complex conditions. The project has the potential to significantly influence scientific understanding and policy development in the field of brain health as it progresses. This Cohort Profile provides the data standardization process and methodology used in the BRIDGE platform, so that many researchers could better understand the BRIDGE platform to optimize their use of the cohort.

## Supplementary Materials

The Supplementary data can be found online at: www.aginganddisease.org/EN/10.14336/AD.2024.1432.
