# Supplementary material for: Profile for Brain Disease Research Infrastructure for Data Gathering and Exploration (BRIDGE) Platform
Source: Aging Dis. 2024 Dec 7;17(1):499–514. doi: 10.14336/AD.2024.1432 (PMC12727072; doi:10.14336/AD.2024.1432)
Supplement: Supplementary file 1 — The Supplementary data can be found online at: www.aginganddisease.org/EN/10.14336/AD.2024.1432. [file AD-17-1-499-s.pdf]

SUPPLEMENTARY DATA

Profile for Brain Disease Research Infrastructure for Data Gathering and Exploration (BRIDGE) Platform

Sujung Oh, Hee-Young Sohn, Junwoo Seo, Eunjee Kang, Jae Kyung Park, So Young Moon, Hee Jin Kim, Na-Yeon Jung, Sun Min Lee, Bo Kyoung Cheon, Hyemin Jang, Sung Hoon Kang, Sarang Kang, Kyu Yeong Choi, Sang-Won Yoo, Yun Joong Kim, Juhee Cho, Eun-Joo Kim, Sang Won Seo, Kun Ho Lee, Joong-Seok Kim, Young Ho Koh, Chi-Hun Kim, Munjin Kwon, Danbee Kang

© 2024. Oh S. et al. Published online at http://www.aginganddisease.org/EN/10.14336/AD.2024.1432

SUPPLEMENTARY DATA

Supplementary Table Cohort 1. Dementia-related cohorts

Type	Name	Participants (N)	Features
Cohort	Alzheimer's Disease Neuroimaging Initiative (ADNI) [1]	3,059	1. Accept 3 lines of application form<br>2. Data release within one day<br>3. Data include expensive and difficulty to access MRI, PET, genetics<br>4. Good QC<br>5. ADNI has also had a great effect in a global sense<br>6. ADNI has resulted in the provision of a large database of images, genetic, fluid biomarker, and clinical data that are being used by many investigators and industry.
	AUSTRALIAN IMAGING, BIOMARKER & LIFESTYLE FLAGSHIP STUDY OF AGING (AIBL) [2]	1,100	1. Data release within one day<br>2. Data include expensive and difficulty to access MRI, PET, genetics<br>3. Volunteers with Alzheimer's disease and mild cognitive impairment (MCI) as well as healthy volunteers.<br>4. The AIBL study was also the first to make use of computerized assessments of cognition in their experimental designs.<br>5. Available on the ADNI LONI website for free download and use by researchers worldwide.<br>6. Data harmonization and sharing: AIBL data have been provided to the Global Alzheimer's Association Interactive Network (GAAIN), and software installed, thereby enabling GAAIN users to interrogate metadata and receive cohort summaries, whereupon users can request further information (and biofluid samples; blood and CSF) if needed by submitting an Expression of Interest (EoI).
	The Australian Dementia Network Registry (ADNeT Registry, or the Registry) [3, 4]	10,000	1. Several dementia CQRs have been established internationally, such as the Swedish Dementia Registry (SveDem), Norwegian Dementia Registry (NorKog), and the Danish Dementia Registry<br>2. Three key pillars within the Australian Dementia Network (ADNeT) initiative:<br>① ADNeT registry: establishing the first dementia clinical quality registry to track, benchmark, and report on the clinical care of people with dementia<br>② ADNeT Memory Clinics: establishing consistent best practice guidelines for the diagnosis and treatment of dementia<br>③ ADNeT Screens and Trials: facilitating the development of effective therapies by providing detailed dementia screening of patients suitable for participation in clinical trials<br>3. Two different recruitment and consent methods (i.e., an opt-out approach and waiver of consent) have been developed based on three key determinants (i.e., capacity to be involved in the opt-out process, person responsible and communication of diagnosis) to ensure that the ADNeT Registry reaches maximum coverage, while respecting patients' choice and privacy.<br>4. A broad definition of 'memory clinic' was used to include all clinicians specialising in dementia diagnosis to gain a broad overview of current clinical practice.
	European Prevention of AD (EPAD) [5]		1. Prospective, multicentre, pan-European longitudinal cohort study with a large sample size recruited mainly from existing parent cohorts.<br>2. Well-phenotyped 'probability-spectrum' population covering the entire continuum of probability for Alzheimer dementia development.<br>3. Readiness population for a Bayesian adaptive designed proof-of-concept trial, with high-quality run-in, pre-randomisation data against which the impact of various interventions will be measured.
	Trial-Ready Cohort for the Prevention of Alzheimer's Dementia (TRC-PAD) [6-8]		1. The partnership between the PI's, an experienced Coordinating Center, the network of sites, academic partners, and the valuable experience and advice of investigators<br>2. Feasible to build a cohort of remotely-consented and enrolled participants with normal cognition, with broad geographic distribution

© 2024. Oh S. et al. Published online at http://www.aginganddisease.org/EN/10.14336/AD.2024.1432

			using an unsupervised cognitive assessment battery to evaluate for increased risk for future cognitive decline.
	Global Alzheimer's Platform (GAP) [9]		1. US-Canada clinical trial network, similar to EPAD<br>2. Scientists learn more about potential racial and ethnic differences in Alzheimer's disease diagnostics and help ensure that the biomarkers developed for Alzheimer's are sensitive and specific for everyone living with the disease.
	Alzheimer's Network (ALZ-NET) [10]		1. Real World Data Platform<br>2. Voluntary provider-enrolled patient registry<br>3. Patients being evaluated for or treated with novel Alzheimer's treatments approved by the FDA in 2021 or after<br>4. Collaborate with affiliated studies conducted by academic, industry, federal or ALZ-NET study teams<br>5. Track health outcomes and resource utilization of participants to inform clinical care
	Brain Health Registry (BHR) [11, 12]	> 100,000 participants	1. BHR approach are scalability and accessibility.<br>2. The online approach permits expanded access to research for individuals who may not be able to participate in in-person studies due to geographic constraints and time burdens.<br>3. Many thousands of participants have returned to the BHR twice per year for many years, providing extensive longitudinal data.
Data Repository	Dementias Platform UK (DPUK) [13, 14]	3,370,929	1. There have DPUK Data portal an it gives researchers anywhere in the world access to high-quality, multi-modal data from more than 50 population and clinical cohort studies, comprising records for over 3.5 million people.<br>2. Free to use<br>3. Requires only internet access<br>4. Ideal for early-career researchers<br>5. 28 day target application response<br>6. Over 40 cohorts<br>7. Secure remote analysis environment<br>8. Pre-installed analytical software<br>9. Wide range of data types including imaging and genetics<br>10. Sophisticated data linkage service
	Global Alzheimer's Association Interactive Network (GAAIN) [14-16]	Cohort and dataset-specific	1. Investigators can address scientific questions of unprecedented complexity by accessing massive shared data sets and can share their own data by joining our global network of Alzheimer's disease study centers.<br>2. The GAAIN Cohort Scout lets you search through thousands of data attributes collected by GAAIN Data Partners and allows you to build, save and share cohorts.<br>3. The GAAIN Interrogator lets you visualize relationships between attributes in user-defined, cross-study cohorts.<br>4. Connect with GAAIN partners to obtain data.<br>5. Save and share your cohort with other
	Alzheimer's Disease Data Initiative (ADDI) [14]	Cohort and dataset-specific	1. The AD Workbench is secure, global, available at no cost, and easy to use.<br>2. It includes secure workspaces, user-friendly workflows, and trusted data governance processes that enable data sharing and foster a cross-domain research environment.<br>3. Upload, combine, curate, harmonize, visualize, and analyze data, then save work in a secure workspace.<br>4. Workspaces can be individual or shared with direct collaborators.
	National Alzheimer's Coordinating Center (NACC) [17]	33 centers and 4 exploratory centers	1. Centralized data repository, and collaboration and communication hub for the National Institute of Aging's (NIA's) Alzheimer's Disease and Research Centers (ADRC) Program<br>2. 42 Alzheimer's Disease Research Centers (ADRCs) throughout the US<br>3. Over the past 20+ years
Data Platform	National Institute on Aging (NIA) [18, 19]		1. Alzheimer's Disease (AD) and AD-Related Dementias (ADRD) Real-World Data Platform

		2. Maximize applicability and generalizability of findings<br>3. Capture more complete information/fill in "gaps" data<br>4. Improve the speed at which scientific questions can be answered<br>7. Improve researcher's ability to answer questions that cannot be feasibly or readily answered via clinical trial<br>8. Facilitate clinical trial recruitment and research participation<br>9. NIA currently has more than 350 clinical trials underway, some of which are testing drug candidates that target many different aspects of the disease, some of which are focused on dementia care and caregiving, and others investigating preventive behavioral and lifestyle interventions.
EPND Cohort Catalogue [20]	(Cohort and dataset-specific)	1. Caregiver and Study Partner Portal<br>2. Co-enrollments with other studies<br>3. Recruitment of underrepresented participant<br>4. Development and validation of online assessment tools<br>5. Data sharing with collaborators and those requesting data<br>6. The Cohort Catalogue is a central, open, accessible repository for researchers to discover ongoing studies and search metadata by disease area, bio sample availability, imaging and cognitive data, and more.
EMIF-AD Catalogue [21, 22]	(Cohort and dataset-specific)	It provides large-scale access to data from more than 50 decentralized Alzheimer's-related studies in Europe.
ROADMAP data cube [23]	(Cohort and dataset-specific)	1. The Cube offers a dynamic overview of the 'landscape' of data availability in Europe for Alzheimer's disease (AD) research.<br>2. The Data Cube combined disparate information from a wide range of European data sources.<br>3. To support the identification and integration of real-world data across multiple sources and countries.<br>4. The Data Cube does provide a clear overview of the data outcomes that are generally available and accessible within Europe.

Supplementary Table 2. Eligible criteria and follow-up schedule for each cohort

	**LEAF cohort**	**LLOD cohort**	**COHD cohort**	**LoPD cohort**
**Eligible criteria**	**Inclusion criteria:**<br>- Patients with early-onset dementia, defined as those with symptoms before the age of 65, including frontotemporal dementia (FTD), Alzheimer's disease-related cognitive impairment (ADCI), and other early-onset neurodegenerative diseases.<br>- Family members of patients with early-onset dementia.<br><br>**Exclusion criteria:**<br>- Patient with a major psychiatric illness other than dementia (schizophrenia, major depression, bipolar	**Inclusion criteria:**<br>Patients aged 65 and older with late-onset dementia, including Alzheimer's disease-related cognitive impairment (ADCI), vascular cognitive impairment (VCI), and Lewy body disease (LBD).<br><br>**Exclusion criteria:**<br>- Patient with neurological abnormalities identified through medical examinations that could cause memory impairment.<br>- Presence of neurological signs indicative of cerebrovascular disease, such as extrapyramidal signs consistent with subcortical lesions.	**Inclusion criteria:**<br>Participants aged 55 years or older.<br><br>**Exclusion criteria:**<br>-Participants with severe hearing, vision, or language impairments that could affect the completion of neuropsychological or other assessments.<br>- History of alcohol addiction, substance abuse, stroke, evidence of central nervous system disorders or damage, epilepsy, depressive disorders, bipolar disorder, schizophrenia, or neurological conditions	**Inclusion criteria:**<br>Patients with Parkinson's disease who visited movement disorder clinics.<br><br>**Exclusion criteria:**<br>Diagnosis of secondary parkinsonism caused by stroke, hydrocephalus, medications, etc.

© 2024. Oh S. et al. Published online at http://www.aginganddisease.org/EN/10.14336/AD.2024.1432

	disorder, alcohol/substance abuse or dependence, delirium, etc.<br>- Presence of brain disease (cerebral infarction, cerebral hemorrhage, brain tumor, etc.) or systemic disease that may significantly affect mental status.<br>- Inability to undergo MRI or CT due to presence of metallic materials in the body, claustrophobia, etc.<br>- Affected by noise on the MRI due to cerebral aneurysm surgery, Ventriculo-Peritoneal Shunt surgery, etc.<br>- Illiteracy to the extent that it would interfere with the performance of neuropsychological testing.<br>- Currently participating in a drug clinical trial<br>- Currently pregnant or lactating<br>- Otherwise deemed unsuitable by the investigator to participate in the study	- History of Axis I psychiatric disorders, including intellectual disabilities, schizophrenia, alcohol dependence, or bipolar disorder.<br>- History of malignant diseases (cancer) within the past 3 years, except for cervical carcinoma in situ or non-melanoma skin cancer.<br>- Have undergone brain surgeries or cerebrovascular surgeries, such as carotid artery surgery or hospitalized due to a head injury.<br>- Experiencing shortness of breath while at rest.<br>- With abnormal blood test results for TSH, folate, vitamin B12, ApoE genotype, or RPR (syphilis test) who continue to show abnormal levels despite treatment.<br>- Experienced memory impairment, language difficulties, or problem-solving disabilities for over two hours following a heart attack, loss of consciousness for more than one hour due to causes other than general anesthesia.<br>- History of hospitalization for mental or emotional disorders, substance abuse, treatment for alcohol dependence within the past 5 years.<br>- Unable to read standard text even with corrective glasses due to impaired vision.<br>- Pregnant or lactating.<br>- Unsuitable for clinical trial participation by the investigator.<br>- Unsuitable for MRI examinations due to conditions such as claustrophobia or the presence of metallic implants.	such as brain infections, Parkinson's disease, multiple sclerosis, cerebrovascular disorders, or cancer.	
**Follow-up**	Every year	Every year	Every year	Every year until 10 years

Supplementary references

[1] Weiner MW, Aisen PS, Jack CR, Jr., Jagust WJ, Trojanowski JQ, Shaw L, et al. (2010). The Alzheimer's disease neuroimaging initiative: progress report and future plans. Alzheimers Dement, 6:202-211.e207.

© 2024. Oh S. et al. Published online at http://www.aginganddisease.org/EN/10.14336/AD.2024.1432

[2] Fowler C, Rainey-Smith SR, Bird S, Bomke J, Bourgeat P, Brown BM, et al. (2021). Fifteen Years of the Australian Imaging, Biomarkers and Lifestyle (AIBL) Study: Progress and Observations from 2,359 Older Adults Spanning the Spectrum from Cognitive Normality to Alzheimer's Disease. J Alzheimers Dis Rep, 5:443-468.
[3] Lin X, Wallis K, Ward SA, Brodaty H, Sachdev PS, Naismith SL, et al. (2020). The protocol of a clinical quality registry for dementia and mild cognitive impairment (MCI): the Australian dementia network (ADNeT) Registry. BMC Geriatr, 20:330.
[4] Mehrani I, Kochan NA, Ong MY, Crawford JD, Naismith SL, Sachdev PS (2021). Organisational aspects and assessment practices of Australian memory clinics: an Australian Dementia Network (ADNeT) Survey. BMJ Open, 11:e038624.
[5] Solomon A, Kivipelto M, Molinuevo JL, Tom B, Ritchie CW (2019). European Prevention of Alzheimer's Dementia Longitudinal Cohort Study (EPAD LCS): study protocol. BMJ Open, 8:e021017.
[6] Walter S, Langford OG, Clanton TB, Jimenez-Maggiora GA, Raman R, Rafii MS, et al. (2020). The Trial-Ready Cohort for Preclinical and Prodromal Alzheimer's Disease (TRC-PAD): Experience from the First 3 Years. J Prev Alzheimers Dis, 7:234-241.
[7] Jimenez-Maggiora GA, Bruschi S, Raman R, Langford O, Donohue M, Rafii MS, et al. (2020). TRC-PAD: Accelerating Recruitment of AD Clinical Trials through Innovative Information Technology. J Prev Alzheimers Dis, 7:226-233.
[8] Aisen PS, Sperling RA, Cummings J, Donohue MC, Langford O, Jimenez-Maggiora GA, et al. (2020). The Trial-Ready Cohort for Preclinical/Prodromal Alzheimer's Disease (TRC-PAD) Project: An Overview. J Prev Alzheimers Dis, 7:208-212.
[9] Dwyer J, Bork J, Zisko L, Goldfeder G, Trotter J, Ritchie CW, et al. (2021). Global Alzheimer’s Platform Foundation® (GAP) development of a transatlantic Alzheimer’s disease clinical trial network. Alzheimer's & Dementia, 17:e052297.
[10] (2022). Alzheimer's Association launches ALZ-NET: A long-term data collection and sharing network for new treatments. Alzheimers Dement, 18:1694-1695.
[11] Weiner MW, Aaronson A, Eichenbaum J, Kwang W, Ashford MT, Gummadi S, et al. (2023). Brain health registry updates: An online longitudinal neuroscience platform. Alzheimers Dement.
[12] Weiner MW, Nosheny R, Camacho M, Truran-Sacrey D, Mackin RS, Flenniken D, et al. (2018). The Brain Health Registry: An internet-based platform for recruitment, assessment, and longitudinal monitoring of participants for neuroscience studies. Alzheimers Dement, 14:1063-1076.
[13] Bauermeister S, Orton C, Thompson S, Barker RA, Bauermeister JR, Ben-Shlomo Y, et al. (2020). The Dementias Platform UK (DPUK) Data Portal. Eur J Epidemiol, 35:601-611.
[14] Toga AW, Phatak M, Pappas I, Thompson S, McHugh CP, Clement MHS, et al. (2023). The pursuit of approaches to federate data to accelerate Alzheimer's disease and related dementia research: GAAIN, DPUK, and ADDI. Front Neuroinform, 17:1175689.
[15] Eckhoff K, Morris R, Zuluaga V, Polsky R, Cheng F (2021). The Association between Tau Protein Level in Cerebrospinal Fluid and Cognitive Status: A Large-Scale Analysis of GAAIN Database. Brain Sci, 11.
[16] Ashish N, Bhatt P, Toga AW (2016). Global Data Sharing in Alzheimer Disease Research. Alzheimer Dis Assoc Disord, 30:160-168.
[17] Monsell SE, Liu D, Weintraub S, Kukull WA (2012). Comparing measures of decline to dementia in amnestic MCI subjects in the National Alzheimer's Coordinating Center (NACC) Uniform Data Set. Int Psychogeriatr, 24:1553-1560.
[18] Hill CV, Pérez-Stable EJ, Anderson NA, Bernard MA (2015). The National Institute on Aging Health Disparities Research Framework. Ethn Dis, 25:245-254.
[19] Ghazarian AL, Haim T, Sauma S, Katiyar P (2022). National Institute on Aging seed funding enables Alzheimer's disease startups to reach key value inflection points. Alzheimers Dement, 18:348-359.
[20] Bose N, Brookes AJ, Scordis P, Visser PJ (2022). Data and sample sharing as an enabler for large-scale biomarker research and development: The EPND perspective. Front Neurol, 13:1031091.
[21] Bos I, Vos S, Vandenberghe R, Scheltens P, Engelborghs S, Frisoni G, et al. (2018). The EMIF-AD Multimodal Biomarker Discovery study: design, methods and cohort characteristics. Alzheimers Res Ther, 10:64.
[22] Konijnenberg E, Carter SF, Ten Kate M, den Braber A, Tomassen J, Amadi C, et al. (2018). The EMIF-AD PreclinAD study: study design and baseline cohort overview. Alzheimers Res Ther, 10:75.
[23] Janssen O, Vos SJB, García-Negredo G, Tochel C, Gustavsson A, Smith M, et al. (2020). Real-world evidence in Alzheimer's disease: The ROADMAP Data Cube. Alzheimers Dement, 16:461-471.

© 2024. Oh S. et al. Published online at http://www.aginganddisease.org/EN/10.14336/AD.2024.1432
